# Lung Adenocarcinoma Harboring *EGFR* Kinase Domain Duplication (*EGFR*-KDD) Confers Sensitivity to Osimertinib and Nivolumab: A Case Report

**DOI:** 10.3389/fonc.2020.575739

**Published:** 2020-12-17

**Authors:** Jie Li, Junrong Yan, Ran Cao, Guanjun Du, Guofang Zhao

**Affiliations:** ^1^ Department of Thoracic Surgery, Hwa Mei Hospital, University of Chinese Academy of Sciences, Ningbo, China; ^2^ Medical Department, Nanjing Geneseeq Technology Inc., Nanjing, China; ^3^ Translational Medicine Research Institute, Geneseeq Technology Inc., Toronto, ON, Canada

**Keywords:** lung adenocarcinoma, targeted next-generation sequencing, *EGFR*-KDD, Osimertinib, Nivolumab

## Abstract

**Background:**

Kinase domain duplication of EGFR (*EGFR*-KDD) is a rare oncogenic driver alteration and serves as a potential therapeutic target. Its effect on EGFR–tyrosine kinase inhibitors (TKIs), especially the third-generation drug Osimertinib, and immune checkpoint inhibitors (ICIs) remains inconclusive.

**Case Presentation:**

A 45-year old male with lung adenocarcinoma progressed with liver metastasis after receiving pemetrexed and cisplatin as adjuvant chemotherapy. Targeted next-generation sequencing (NGS) identified an *EGFR*-KDD in the resected left upper lung. Icotinib was used in the following treatment and the liver metastasis was found to shrink but the progression-free survival (PFS) only lasted for 4 months with the appearance of right hepatic metastasis. Meantime, the same *EGFR*-KDD was identified in the left hepatic re-biopsy. Afterward, the patient benefited from the third-line therapy of Osimertinib with a PFS as long as 21 months. Then he progressed with enlarged mediastinal lymph nodes, and targeted NGS consistently identified *EGFR*-KDD, as well as a new *RELN* p.G1774E mutation. Given the continually increasing tumor mutation burden (TMB, 3.4 mutation/Mb) and PD-L1 expression-based tumor proportion score (TPS, 1%), Nivolumab was used as the fourth-line salvage therapy, which lead to considerable efficacy, with decreased blood carcinoembryonic antigen (CEA), regressed mediastinal lymph nodes, and reduced liver metastases.

**Conclusions:**

Our case provided direct evidence to support the role of Osimertinib in the treatment of *EGFR*-KDD, as well as added valuable insights into application of immune-based therapeutics in the specific subgroups bearing *EGFR* alteration(s).

## Background

The discovery of oncogenic aberrations in epidermal growth factor receptor (*EGFR*), which commonly occur as 19 exon deletion or L858R mutation, boosts the treatment of targeted therapy in non-small cell lung cancer (NSCLC). As a rare *EGFR* alteration, kinase domain duplication (KDD), firstly identified as a driver aberration and therapeutic target in 2015, is an in-frame duplication in exons that encode the EGFR tyrosine kinase domain (1). The current reported prevalence of *EGFR*-KDD in NSCLCs is 0.04% (2) in European and American and 0.07% (3)~0.12% (4) in East Asian patients, respectively. When with this rare aberration, the response to EGFR-tyrosine kinase inhibitor (TKI) and immune checkpoint inhibitors (ICIs) remains inconclusive. Here we described a case with advanced lung adenocarcinoma harboring *EGFR*-KDD who achieved differentiate response to first and third generation EGFR-TKIs as well as programmed death receptor-1 (PD-1) inhibitor Nivolumab.

## Case Presentation

**Figure 1:**
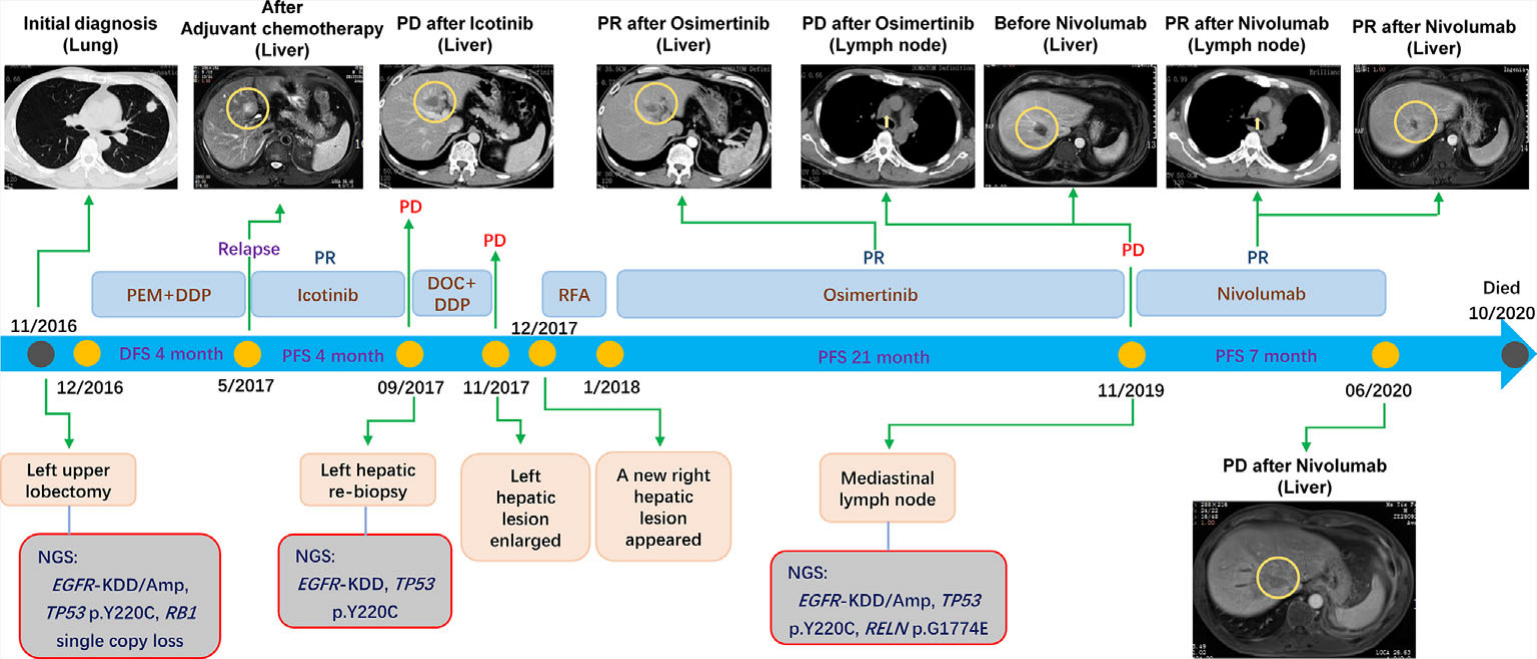
The timeline showing the history of treatment and examinations for the patient under current study.

A 45-year-old male underwent a left upper lobectomy and postoperative pathology revealed invasive stage IIIA lung adenocarcinoma (**Figure 1**). Targeted next- generation sequencing (NGS) with a customized panel (Geneseeq Prime panel) designed to target 425 cancer-specific genes was performed, and four somatic mutations and copy number alterations (CNAs) were identified, including *EGFR*-KDD of exon 18-25 [mutant allele frequency (MAF): 13.5%], *EGFR* amplification (4.5-fold), TP53 p.Y220C (MAF: 37.0%), and *RB1* single copy loss (**Figure 1**). The tumor mutation burden (TMB) was estimated to be 1.1 mutation/Mb. The patient received pemetrexed and cisplatin as adjuvant chemotherapy. Four months later, he progressed with liver metastasis in left lobe (**Figure 1**).

Then, the patient was treated with Icotinib and the metastasis shrunk. Unfortunately, the drug resistance was observed only after 4 months, as evidenced by the fact that previously responsive liver lesion progressed. Left hepatic re-biopsy confirmed metastatic adenocarcinoma and target sequencing (Geneseeq Prime panel) detected the same *EGFR*-KDD (MAF: 4.9%) as well as mutation of *TP53* p.Y220C (MAF: 0.5%) (**Figure 1**). The TMB was calculated as 2.2 mutation/Mb.

Docetaxel and cisplatin were initiated as the second-line therapy. However, the left hepatic metastasis enlarged rapidly after 2 cycles of chemotherapy. The blood carcinoembryonic antigen (CEA) level increased from 9.5 mg/ml (before chemotherapy) to 22.7 mg/ml. Even worse, a right hepatic metastasis appeared soon afterward. Radiofrequency ablation (RFA) of liver was conducted on both of the left and right hepatic metastases, but no reduction in liver lesions was observed, and the CEA level showed a slight increase from 7.3 to 10.3 mg/ml.

Afterward, the patient started taking Osimertinib (80 mg once daily). Encouragingly, both liver lesions showed significant regression (**Figure 1**). One month after initiation of Osimertinib, the CEA level decreased to 5.4 mg/ml, and remained at normal level for 18 months. Moreover, the progression-free survival (PFS) reached 21 months. However, the CEA level increased to 23.1 mg/ml at the 19^th^ month after the initiation of Osimertinib treatment, and 2 months later, the patient progressed with enlarged mediastinal lymph nodes (**Figure 1**) with the CEA level of 73.9 mg/ml. Resampling and targeted sequencing (Geneseeq Prime panel) consistently identified *EGFR*-KDD (MAF: 33.9%), as well as *EGFR* amplification (6.6-fold), *TP53* p.Y220C (MAF: 53.3%), and a new mutation of *RELN* p.G1774E (MAF: 45.4%) (**Figure 1**). The estimated TMB increased to 3.4 mutation/Mb. In addition, the assessment of PD-L1 expression using antibody 28-8 (pharm Dx, Dako’s Platform) showed tumor proportion score (TPS) of 1%.

On these bases, the fourth-line salvage therapy using Nivolumab was prescribed and the therapeutic efficacy was considerable, as evidenced by the decreased CEA, regressed mediastinal lymph nodes, reduced metastases in both left and right liver (**Figure 1**). Specifically, the CEA level decreased from 143.6 to 41.8 mg/ml one month later. The PFS reached 7 months and no obvious adverse effects were observed. The quality of life was in good status during the Nivolumab treatment. After that, the patient progressed with enlarged liver metastasis. Unfortunately, the patient was also infected with tuberculosis, and his condition took a sharp turn for the worse due to both tumor progression and tuberculosis. The families gave up further treatment and the patient died 4 months later.

## Discussion

Classical *EGFR* alterations confer continual activation of protein kinase function and sensitivity to EGFR TKI (5). As a rare oncogenic variant, *EGFR*-KDD is able to form asymmetric homo-dimer and thus activate *EGFR* signaling pathway (1). Several pilot studies confirmed the effectiveness of EGFR-TKIs in NSCLCs harboring *EGFR*-KDD (1, 3, 4, 6–9) (**Table 1**). In our case, the patient bearing *EGFR*-KDD was sensitive to Icotinib and Osimertinib with PFS of 4 and 21 months, respectively. According to previous reports, there are greatly varying efficacies across the first-generation TKIs against *EGFR*-KDD, among which the longest PFS up to 6 years was achieved by Gefitinib (6). In our case, a PFS of only 4 months was observed on Icotinib treatment. In comparison, the third-generation TKI Osimertinib presented an encouraging PFS as long as 21 months. The mechanism underlying such difference in the clinical outcomes is worth investigation. Most recently, our group conducted a molecular dynamics simulation-guided study of *EGFR*-KDD effect on different TKIs (10). It was shown that Gefitinib, as the first-generation EGFR-TKI, suffered from more disturbances in the *EGFR*-KDD binding event than the third-generation EGFR-TKI, Osimertinib. Moreover, Osimertinib was found with higher binding affinity toward *EGFR*-KDD than Gefitinib. These results provide the structural basis of evidence that Osimertinib, compared to the first generation TKI, is able to bind and thus inhibit *EGFR*-KDD with more potency.

**Table 1:** Summary of response to EGFR-TKIs in NSCLCs harboring EGFR-KDDs.

Study	Population	Best response to TKIs	TKIs, response and PFS
Our case	East Asian	PR	Icotinib, PR, 4m; Osimertinib, PR, 21m
Gallant et al. (1)	American	PR	Afatinib, PR, 10m
Baik et al. (6)	American	PR	Gefitinib, PR, 6y; Erlotinib, PR, 3y
Wiest et al. (7)	Germany	PR	Afatinib, PR, NA
Zhu et al. (8)	East Asian	SD	Icotinib, SD, 11m (Not reach)
Xu et al. (9)	East Asian	PR	Afatinib, PR, NA
Wang et al. (3)	East Asian	SD	Icotinib, SD, NA
Wang et al. (4)	East Asian	PD	Erlotinib, PD, 2m; Osimertinib, PD, 2m
	East Asian	PR	Gefitinib, PR, 5m; Afatinib, PD, 2m Osimertinib, PR, 4m (Not reach)
	East Asian	SD	Gefitinib, SD, 11m
	East Asian	PR	Icotinib+apatinib, PR, 4m (Not reach)
	East Asian	PD	Gefitinib, PD, 3m Erlotinib, PD, 5m

ICIs serve as a new standard of care for advanced NSCLCs with no *EGFR* mutation. However, the study concerning the therapeutic effect of ICIs on *EGFR* mutant lung cancer is sparse and the outcome seems not optimistic. Previous evidence showed that compared with chemotherapy, there was no superiority in terms of overall survival (OS) when ICIs were used as the second line treatment among *EGFR*-mutant subgroup (11). The Atlantic trial demonstrated the overall response rate (ORR) of ICIs was 9.8% with averaged PFS of only 1.9 months among *EGFR*+/*ALK*+ individuals (12). Cho et al. also suggested *EGFR* mutant NSCLC patients benefited less from ICIs treatment (13). Similar results were found in Italian Nivolumab expanded access program, which showed ORR of 8.8% among *EGFR* mutant subgroup (14). Consistently, a retrospective study by Hastings et al., concluded with an ORR of 9.9% for ICIs treatment in *EGFR*-mutant NSCLCs (15). Despite these, it is worth to mention that adding atezolizumab to standard-of-care Bevacizumab and chemotherapy increased PFS and OS benefit among the *EGFR*-mutant patients (16).

Of note, *EGFR* aberrations were found to be corelated with significantly increased rate of tumor growth after ICIs monotherapy (17). Pilot study suggested that *EGFR* pathway activation resulted in a signature of immuno-suppression, driving immune escape (18). Furthermore, certain *EGFR* aberrations, including *EGFR* 19Del and T790M, are considered to be related to ICIs-induced hyper-progressive disease (HPD). Recently, our group reported a patient with *EGFR* 20 exon insertion and MYC amplification who suffered from HPD after treatment of Nivolumab, resulting in rapid death in 2 months (19). *Ex vivo* study exhibited that PDX model carrying *EGFR* 21 exon L858R mutation also mirrored the clinical observation of HPD following Nivolumab treatment (20). Here, our patient significantly benefited from ICIs treatment in the presence of *EGFR*-KDD. Emerging evidence showed *EGFR* 20 exon insertion mutation tended to present higher PD-L1 expression than classic *EGFR* mutation, and in turn, was related with improved outcome in response to ICIs (21). Case series showed patients harboring *EGFR* G719X mutations along with high PD-L1 expression conferred sensitivity to ICIs-based treatment (22). The aforementioned Hastings’ study (15) further investigated the efficacy differences between various *EGFR* subtypes. Therapeutic efficacy was best for *EGFR* G719 but worst for *EGFR* L861Q. For common mutant subgroups, *EGFR* 19Del showed worse response than *EGFR* L858R. On contrary, negative association between *EGFR* alteration and HPD was observed from two independent cohorts (23, 24). These data suggest the responsiveness to ICIs in patients with *EGFR* aberrations may differ in terms of specific aberrant type. To overcome the low response rates to PD-1 pathway blockade, highly specific patient(s) with *EGFR*-driven tumor should be screened out for ICIs monotherapy and combinations.

There are several limitations in the present study. Owing to the coverage of currently used sequencing panel, it was not available to explore the molecular basis of mechanism underlying the drug resistance observed in the clinic, e.g., Icotinib and Osimertinib. According to previous studies, there exist varying conclusions as to the efficacies of the first-generation EGFR-TKIs in the treatment of *EGFR*-KDD, as well as the uncertain response to ICIs among *EGFR* mutant tumors. In this context, our current case report only provided an example but not guidance for the clinical intervention, which clearly demands more extensive investigations.

Collectively, our case provides direct evidence to support the role of Osimertinib in the treatment of *EGFR*-KDD, as well as added valuable insights into application of immune-based therapeutics in the specific subgroups bearing *EGFR* alteration(s).

## Data Availability Statement

The original contributions presented in the study are included in the article/**Supplementary Material**. Further inquiries can be directed to the corresponding author.

## Ethics Statement

The studies involving human participants were reviewed and approved by Ethics Committee of Hwa Mei Hospital, University of Chinese Academy of Sciences. The patients/participants provided their written informed consent to participate in this study. Written informed consent was obtained from the individual(s) for the publication of any potentially identifiable images or data included in this article.

## Author Contributions

GZ designed the entire study. JL carried out patient clinical management and sample collection. JY, RC, and GD analyzed the data. JL and JY wrote the manuscript. All authors contributed to the article and approved the submitted version.

## Funding

This work was supported by grant from Hwa Mei Research Foundation of 2016 (2016HMKY05).

## Conflict of Interest

JY, RC, and GD were employed by the company Geneseeq Technology, Inc.

The remaining authors declare that the research was conducted in the absence of any commercial or financial relationships that could be construed as a potential conflict of interest.
